# The Impact of Vitamin D Deficiency on Retinopathy and Hearing Loss among Type 2 Diabetic Patients

**DOI:** 10.1155/2018/2714590

**Published:** 2018-07-09

**Authors:** Abdulbari Bener, Mustafa Eliaçık, Hakan Cincik, Mustafa Öztürk, Ralph A. DeFronzo, Muhammad Abdul-Ghani

**Affiliations:** ^1^Department of Biostatistics & Medical Informatics, Cerrahpaşa Faculty of Medicine, Istanbul University, Istanbul, Turkey; ^2^Department of Evidence for Population Health Unit, School of Epidemiology and Health Sciences, The University of Manchester, Manchester, UK; ^3^Department of Endocrinology, Medipol International School of Medicine, Istanbul Medipol University, Istanbul, Turkey; ^4^Department of Ophthalmology, Medipol International School of Medicine, Istanbul Medipol University, Istanbul, Turkey; ^5^Department of ENT and Audiology, Medipol International School of Medicine, Istanbul Medipol University, Istanbul, Turkey; ^6^Division of Diabetes, University of Texas Health Science Center at San Antonio, 7703 Floyd Curl Drive, San Antonio, TX 78229, USA; ^7^Academic Health System, Hamad General Hospital, Doha, Qatar

## Abstract

**Aim:**

The current study was aiming to investigate the relation between vitamin D, retinopathy, and hearing loss among type 2 diabetes mellitus (T2DM) patients.

**Methods:**

Cross-sectional study carried on 638 subjects aged between 20 and 60 years who visited the Endocrinology, Ophthalmology, and ENT Outpatient Clinics of the Medipol Hospital during the period from March 2016 to May 2017. Two audiometers Grason Stadler GSI 61 and Interacoustics AC40 Clinical audiometer were used to evaluate the hearing loss. Risk factors for diabetic retinopathy were evaluated, including age, sex, diabetes duration, glycated hemoglobin (HbA1c), hypertension, and lipid profiles.

**Results:**

The mean age (± SD, in years) for retinopathy with hearing loss versus normal subjects was 47.7 ±10.2 versus 48.5±9.1. The associated risk factors were significantly higher in T2DM with hearing loss, hypertension (32.6% versus 15.7%), tinnitus (40.0% versus 18.0%), vertigo (59.7% versus 26.8%), and headache (54.9% versus 45.3%), than in normal hearing diabetes. There were statistically significant differences between hearing impairment versus normal hearing for vitamin D [19.40±9.71 ng/ml versus 22.67±9.28 ng/ml; p<0.001], calcium, magnesium, phosphorous, cholesterol, HDL-C, LDL-C, albumin, systolic blood pressure [131.70±9.25 Hg versus 127.73±11.98 Hg], diastolic blood pressure [82.20±8.60 mm Hg versus 79.80±8.20 mm Hg], and microalbuminuria. Multivariate logistic regression analysis revealed that variables such as vertigo, duration of DM, mobile/I pad phone, vitamin D deficiency, sleeping disturbance, headache, frequently TV watching, tinnitus, cigarette smokers, and hypertension were considered at higher risk as a predictors of retinopathy with hearing loss among diabetic patients.

**Conclusion:**

Vitamin D deficiency is considered as a risk factor for diabetic retinopathy and hearing loss among diabetic patients. Meanwhile, hyperglycemia could be considered as a modifiable risk factor for diabetic retinopathy; tight glycemic control may be the most effective and important therapy for improving quality of life and substantially reducing the incidence of retinopathy and in T2DM patients.

## 1. Introduction

Type 2 diabetes mellitus (T2DM) is a major health problem associated with significant morbidity, mortality, and healthcare expenditure. The etiology of T2DM is complex and includes abnormalities in multiple organs, including liver, skeletal muscle, adipocytes, gut, and beta cell [1, 2]. T2DM also is a major risk factor for cardiovascular disease [3, 4] which is the leading cause of death in western countries [5, 6]. Several modifiable lifestyle factors, e.g., sleep duration, physical activity, and healthy-balanced diet can reduce the incidence of T2DM among high risk individuals [4, 5]. The prevalence of T2DM is even more widespread in the Middle East and its impact on public health is greater than other regions of the world [1, 2]. According to a population-based study in Turkey, the prevalence of diabetic retinopathy was 12.8% among type 2 diabetic patients [4]. Diabetic retinopathy has a complex process [4–6].

Recent studies have reported that vitamin D deficiency is closely related to obesity and increased risk of T2DM [6–12]. There are very strong correlations between vitamin D status, obesity, and T2DM [5–7, 12–14]. Similarly, several authors found that vitamin D deficiency is a predisposing factor for developing diabetes and increasing hearing loss [6–15]. Vitamin D insufficiency is very common worldwide. Different epidemiological studies have reported that more than 40% of adult populations are at risk of vitamin D insufficiency [5]. Further, vitamin D deficiency may play a role in the pathogenesis of diabetic retinopathy [6, 7, 13, 16]. Thus, it is not surprising that hearing loss if often found in T2DM patients [6, 7, 12, 14–17]. A number of studies have attempted to identify the cause of hearing loss in T2DM [6–11, 13] and found hyperglycemia to be an important risk factor. Because hyperglycemia is the principal risk factor for diabetic microvascular complications, i.e., retinopathy, neuropathy, and nephropathy [5–7, 14], we hypothesize that a strong correlation between hearing loss and diabetic retinopathy will be present in T2DM patients [3, 6, 8, 9, 16]. Because low level of vitamin D was also related to increased risk of hearing loss and retinopathy, we also hypothesized that T2DM subjects who manifest hearing impairment and retinopathy may also have low plasma vitamin D levels [5–7, 13]. The aim of this study was to investigate the relationship between vitamin D, retinopathy, and hearing loss among T2DM.

## 2. Subjects and Methods

This is a cross-sectional study conducted on patients aged from 20 and 60 years who visited the Endocrinology, Ophthalmology, and ENT Outpatient Clinics of the Medipol Hospital during the period from March 2016 to May 2017. IRB ethical approval for this study was obtained from the Medipol International School of Medicine, Istanbul Medipol University, and informed written consent was obtained from patients before the start of the study.

The present sample was based on the representative registered 900 patients with diagnosed diabetes. Of the 900 approached registered patient with T2DM, 638 (70.9%) agreed to participate in this study at the Medipol International School of Medicine, Istanbul Medipol University.

### 2.1. Laboratory Measurements

The patients were considered to have DM if they have a history of DM and are currently taking oral medications for diabetes; we have followed the methods described by Bener et al. 2017 [1]; American Diabetes Association [3] and International Diabetes Federation (IDF) [2]; Bener et al. [1, 6]. Meanwhile, DM has been defined when fasting venous blood glucose concentration is equal to or higher than 7.0mmol/L and/or for a 2-hour postglucose tolerance test (GTT); venous blood glucose concentration is higher than 11.1 mmol/L. Oral glucose tolerance test (OGTT) was carried out only if blood sugar was less than 7 mmol/l. The inclusion criteria are comprised of diagnosis of T2DM according to IDF criteria international standards by WHO and IDF [2, 3], fasting plasma glucose (FPG) higher than 7.0 mmol/L and/or 2-hour postprandial plasma glucose (PPG), or random plasma glucose higher than 11.1 mmol/L [5]. Furthermore, having regular antidiabetic drug treatment for at least 3 years, aged between 20 and 60 years, residence in a city of Istanbul for more than 3-year period.

The study included sociodemographic characteristics, lifestyle habits, BMI, comorbid symptoms, diabetic complications, systolic and diastolic blood pressures, clinical biochemistry serum triglyceride, total cholesterol, high-density lipoprotein (HDL-C) cholesterol, low density lipoprotein (LDL-C) cholesterol, hemoglobin A1c (HbA1c), and fasting glucose levels (FPG) which were collected. Hypertension criteria are defined according to the World Health Organization (WHO) [18] where systolic blood pressure (SBp) ≥ 140 mmHg or Diastolic Blood Pressure (DBp) ≥90 mmHg or using antihypertensive medication [18].

#### 2.1.1. Ophthalmic Assessment Method

All the patients with diabetes underwent a complete ophthalmic examination comprised of best-corrected visual acuity, indirect ophthalmoscopy, slit-lamp biomicroscopy, and fundus fluorescein angiography. ETDRS (Early Treatment Diabetic Retinopathy Study) [1] adaptation of the modified Airlie House classification system was used for diabetic retinopathy grading and diabetic retinopathy was further categorized as nonproliferative diabetic retinopathy (NPDR) and proliferative diabetic retinopathy (PDR). The ophthalmologist at the reference center inspected the downloaded digital images, evaluated them for the presence or absence of diabetic retinopathy, and graded the retinopathy using a modified version of the Airlie House classification [19].

#### 2.1.2. Hearing Assessment Method

Pure tone audiometry is a behavioral test used to measure hearing sensitivity. This measure involves the peripheral and central auditory systems [5, 15, 20]. Two clinical digital audiometers (Garson Stadler GSI 61 and Interacoustics AC40 Clinical Audiometer, Interacoustics, Assens, Denmark) are used to be a device for diagnosing hearing loss that are regularly calibrated to international standards which were performed by pretrained technicians to test patients' hearing level. Pure tone audiometry was measured at 0.25, 0.5, 1, 2, 4, and 8 kHz to detect the hearing threshold at each given frequency in a sound-isolated room, standardized according to the manufacturer's instructions. Pure tone average (PTA) was determined, based on the air-conduction average threshold levels in each ear (in decibels) at 0.5, 1, 2, and 4 kHz. This pure tone average is an indication of how well a patient can hear a normal conversation. Patients without hearing loss would have a 0 to 26 dB loss. Hearing loss evaluation is described as follows [5, 15, 20]: normal (≤ 26 dB) and 26 dB above, hearing loss.

The statistical analysis was performed by using the Statistical Package for Social Sciences [SPSS]. Student's t-test was used to ascertain the significance of differences between mean values of two continuous variables and confirmed by the nonparametric Mann-Whitney tests to determine if the results differed, indicating lack of normal distribution of the variable. Chi-square test Fisher's exact test (two-tailed) were used to test significance differences between two or more categorical groups. A multivariate logistic regression model was performed to assess the relation between selected lifestyle factors, retinopathy, and presence of hearing loss. Confounders were assessed statistically through change in beta coefficient (crude *β*-adjusted *β*), and if the change was more than 10%, the variable was considered as a confounder and retained in the final model. The cut-off value for significance was chosen as 0.05.

## 3. Results

Table 1 presents the sociodemographic characteristics of subjects with hearing impairment and subjects with normal hearing. Majority of the hearing loss was observed at the age above 45 years of age. More than 90% of the studied patients were frequent users of mobile phones and approximately 88% of subjects with hearing loss were watching TV. The mean duration of diabetes was 9.54±5.31 years, duration of sleep was 5.88±1.25 hours, and 27.8% had positive family history of diabetes. Subjects with hearing impairment had worst risk factor profile than T2DM patients with normal hearing: hypertension, (23% versus 14.8%), tinnitus (33.3% versus 21.1%), vertigo (25.4% versus 16.6%), and headache (54.0% versus 42.0%), respectively (Table 1).

Table 2 presents the metabolic characteristics of the two groups. Vitamin D levels were significantly lower in subjects with hearing impairment compared to subjects with normal hearing [19.40±0.71 ng/ml versus 22.65±9.280 ng/ml; respectively, p<0.001]. Other parameters were calcium [2.18±0.38 ng/ml versus 2.39±0.26 mmol/L; p<0.001], magnesium [0.76±0.10 mmol/L versus 0.90±0.09 mmol/L; p<0.001], phosphorous [1.40±0.32 mmol/L versus 1.58±0.29 mmol/L; p<0.001], creatinine [68.74±14.47 mmol/L versus 66.69±17.81 mmol/L; p=0.007], cholesterol [3.40±0.66 mmol/L versus 3.16±0.80 mmol/L; p=0.020], HDL(1.45±0.90 mmol/L versus 1.22±0.37 mmol/L; p=0.001], LDL [1.97±0.92 mmol/L versus 1.72±0.69 mmol/L; p=0.025], albumin [40.10±4.41 mmol/L versus 38.68±4.09 mmol/L; p=0.001], systolic blood pressure [131.70±9.25 mmHg versus 127.73±11.98 mmHg; p=0.017], and diastolic blood pressure [82.20±8.60 mmHg versus 79.80±8.20 mmHg; p=0.004] and microalbuminuria [14.59±2.49 mmol/L versus 9.07±3.50 mmol/L p<0.001].

Table 3 presents the mean distribution of Pure Tone Thresholds for both groups, with retinopathy and hearing loss and without retinopathy and normal hearing. There were highly statistically significant differences between right and left ear frequency in dB unit (p<0.001). The mean and St. Dev of PTA in the right ear for retinopathy with hearing impairment was 33.21±14.55 dB, compared to 19.34±10.42 dB in the nonretinopathy with normal hearing. The mean and St. Dev of PTA in the left ear for retinopathy with hearing impairment was 32.07±10.11 dB compared to 19.07±12.7 dB in nonretinopathy with normal hearing. Differences in speech reception threshold [SRT] between the two groups were also significant in both the right and left ear (p<0.001). Also, the speech discrimination score [SDS] as nonretinopathy with normal hearing had better SDS than the retinopathy with hearing impairment; the differences were highly significant for either the right or left ears (p<0.001).

Meanwhile, in present study, we have identified a significant strong correlation coefficient between diabetic retinopathy and SBp (r= 0.439, p= 0<0.01) DBp (r= 0.226, p= 0<0.01), HbA1c (r= 0.446, p= 0<0.01), BMI (r= 0.233, p= 0<0.01), and physical activity (r= 0.126, p= 0<0.05).

Table 4 presents multivariable logistic regression analysis of variables for predictors of hearing loss with neuropathy among diabetic patients. Vertigo (OR 4.09 95% CI 1.79-9.66; p<0.001), duration of DM (OR 1.92 95%CI 1.70-2.44; p<0.001), mobile/I pad phone (OR 4.25 95% CI 2.85-9.97; p<0.001), vitamin D deficiency (OR 2.03; 95%CI 1.94-3.06, p= 0.002), sleeping disturbance (OR 2.68; 95%CI 2.24-3.58, p=0.004), headache (OR 2.39 95%CI 1.30-4.32; p=0.005), frequently TV watching (OR 2.42 95%CI 1.26-4.00; p=0.006), tinnitus (OR 1.95 95%CI (1.22-3.13); p= 0.007), cigarette smokers (OR 1.79; 95%CI 1.26-3.23, p=0.029), and hypertension (OR 1.71 95%CI 1.36-2.82); p= 0.036) were considered at higher risk as a predictors of retinopathy with hearing loss among diabetic patients.

## 4. Discussion

The results of the present study have revealed high prevalence of hearing impairment in association with retinopathy in T2DM patients. The association between diabetes and eye disease is well recognized with diabetic retinopathy as the most common eye disease in subjects with T2DM [9]. However, the link between diabetes and hearing loss has not been universally accepted until recently. The first positive correlation was found in 1961 by Jorgensen and Bush [20] which showed that subjects with proliferative diabetic retinopathy were twice as likely to have hearing loss. However, more recent studies agree that diabetes is correlated with high-frequency hearing loss [9].

More recently, Bener et al. [5, 6] studied 1,633 diabetic patients and reported that an overall prevalence of peripheral neuropathy was 9.5% among them. The analysis showed that the condition was significantly associated with age, male gender, consanguinity, family history of DM, and elevated blood pressure. More recently from a study by Ooley et al. [9] after controlling for glucose control, as measured by HbA1c and renal function measured with serum creatinine, the presence of diabetic retinopathy was significantly associated with the severity of hearing loss in both ears (right ear, P = .018 and left ear, P = .007). Usually, the chronic hyperglycemia in diabetic patients could affect hearing leading to hearing impairment. Several studies have reported close association between hearing impairment and DM (3,9,12). Furthermore, more recently Bener et al. [1] and Ooley et. all [9] reported that HbA1c level was associated with hearing impairment in nondiabetic participants, and higher HbA1c levels could be used as an indicator for the presence of hearing loss. A recent meta-analysis concluded that mild hearing loss is more prevalent in participants with DM [11].

The current study results are consistent with previous reports about the close relationship between T2DM and high-frequency sensorineural hearing loss [1, 8–12, 20, 21] and impaired auditory brainstem responses [7, 10]. These results are in support of the fact that diabetes mellitus may have very complex repercussion on the auditory pathways. The correlation between hearing loss and hypertension can be considered as an important risk factors. Most previous studies reported that hypertension was associated with high-frequency hearing impairment, and likewise, in this study, we found a positive association between these variables [6, 9, 20, 21].

More recent study by Praidou et al. [22] reported reduced total physical activity in patients with severe to very severe nonproliferative diabetic retinopathy and proliferative diabetic retinopathy compared to patients with mild to moderate nonproliferative diabetic retinopathy and to control group. Also, the current study showed the a significant strong association between diabetic retinopathy and some risk factors such as SBp, DBp, HbA1c, BMI, and physical activity. Furthermore, our results also are in an agreement with previous suggestion more recently by Bener et al. [1] and Praidou et al. [22] about the correlation between HbA1c levels, physical activity, BMI, and diabetic retinopathy.

The current study reveals strong association between high prevalence of hearing impairment, vertigo, and tinnitus in T2DM patients [1, 5, 6]. The results of this study demonstrated a strong correlation between glucose control as measured by HbA1c, serum vitamin D levels, the degree of diabetic retinopathy, and the severity of sensorineural hearing loss which is confirmative most recently study by Bener et al. [1] with diabetic neuropathy.

The present study is not without its limitations. Firstly, this is a cross-sectional design with limits the ability to assess causality. Secondly, only one time point was recorded for the subjects in this study. Thirdly, also, there is possible selection bias as this is not a study of consecutive patients seen at our institution. Although, dietary intake and outdoor exposure data were collected. Additionally, effects from sunlight exposure were minimized in this study as all subjects were enrolled over a twelve-month period. Finally, beside several limitations, the analysis revealed that the overall mean standard deviation of 25(OH)D in this study was statistically strong significant differences between hearing impairment versus normal hearing for vitamin D [19.40±0.71 ng/ml versus 22.65±9.280 ng/ml; p<0.001].

## 5. Conclusion

The current study results suggests a strong positive association between vitamin D, retinopathy, and hearing impairment among T2DM. Vitamin D deficiency is an independent risk factor for diabetic retinopathy and hearing loss among diabetic patients. Meanwhile, hyperglycemia could be considered as a modifiable risk factor for diabetic retinopathy; tight glycemic control may be the most effective and important therapy for improving quality of life and substantially reducing the incidence of retinopathy and in T2DM patients.

## Figures and Tables

**Table 1:** Comparison of sociodemographic and clinical characteristics between diabetic retinopathy patients with and without hearing loss (N=638).

**Variables**	**Retinopathy with ** **Hearing Loss ** **≥26 dB n=126**	**Non-Retinopathy ** **Normal Hearing ** **<26 dB n=512**	**Odds Ratio ** **95%CI**	**P value**
**Age groups (in years):**				
<40	21(16.7)	160(31.2)	1	
40-49	32(25.4)	115(22.5)	2.12(1.16-3.86)	0.014
50-59	24(19.0)	122(23.8)	1.50(0.79-2.81)	0.208
60 and above	49(38.9)	115(22.5)	3.24(1.18-4.08)	<0.001
**Gender:**				
Male	54(42.9)	178(34.8)	1.50(0.94-2.09)	0.091
Female	72(57.1)	334(65.2)	1	
**Level of education:**				
Intermediate	56(44.4)	183(23.8)	1.50(1.00-2.47)	0.050
Secondary	31(24.6)	129(26.4)	1.23(0.73-2.07)	0.431
University	39(31.0)	200(37.6)	1	
**BMI (kg/m** ^**2**^ **):**				
Normal (<25 kg/m^2^)	33(26.2)	138(27.0)	1	
Overweight 25-30 kg/m^2^	52(41.3)	238(46.50)	0.91(0.56-1.48)	0.714
Obese >30 kg/m^2^	41(32.5)	136 (26.6)	1.26(0.75-2.11)	0.378
**Physical activity:**				
More than 30 min/day	38(30.2)	144(28.1)	1	
Less than 30 min/day	88(69.8)	368(71.9)	0.90(0.59-1.38)	0.650
**Fast food habits:**				
Yes	31(24.6)	76(14.8)	1.50(0.94-2.41)	0.009
No	95(75.4)	436(75.2)	1	
**Cigarette Smoking status:**				
Yes	27(21.4)	69(13.5)	1.75(1.16-2.87)	0.026
No	99(78.6)	443(86.5)	1	
**Do you use mobile phone**				
Yes	130(90.3)	423(81.8)	1.95(1.08-3.54)	0.027
No	14(9.7)	89(18.2)		
**Can you watch/hear TV:**				
Yes	111(88.1)	394(77.0)	2.21(1.24-3.94)	0.006
No	15(11.9)	118(23.0)	1	
**Family history of DM:**				
Yes	35(27.8)	83(16.2)	1.98(1.26-3.13)	0.003
No	91(71.2)	429(83.8)	1	
**Hypertension**				
Yes	29(23.0)	76(14.8)	1.71(1.10-2.77)	0.028
No	97(77.0)	436(85.2)	1	
**Tinnitus**				
Yes	42(33.3)	108(21.1)	1.87(1.22-2.87)	0.004
No	84(66.7)	404(78.9)	1	
**Vertigo**				
Yes	32(25.4)	85(16.6)	1.71(1.08-2.71)	0.023
No	94(74.6)	427(83.4)	1	
**Headache**				
Yes	68(54.0)	215(42.0)	1.61(1.09-2.40)	0.044
No	58(46.0)	297(58.0)	1	
**Duration of diabetes (years)**	9.54±5.31	8.19±4.42	1.64(1.14-2.36)	0.028
**Sleeping (in hrs) **	5.88±1.25	6.39±1.24	2.93(1.96-4.45)	0.001

**Table 2:** Clinical biochemistry baseline value among retinopathy hearing loss and normal hearing subject among T2DM patients (N = 638).

**Variables **	**Retinopathy with ** **Hearing Loss ** **≥26 dB n=126 ** **Mean ± SD**	**Non-Retinopathy ** **Normal Hearing with ** **diabetic <26 dB ** **N = 512** **Mean ± SD**	**P value**
Vitamin D (ng/ml)	19.40±9.71	22.67±9.28	<0.001
Hemoglobin (g/dL)	12.85± 2.40	12.98±2.35	0.285
Magnesium (mmol/L)	0.76±0.10	0.90±0.09	<0.001
Potassium (mmol/L)	4.49±0.45	4.48±0.38	0.830
Calcium (mmol/L)	2.18±0.38	2.39±0.26	0.036
Phosphorous (mmol/L)	1.40±0.32	1.58±0.29	<0.001
Creatinine (mmol/L)	68.74±14.47	66.69±17.81	0.231
Fasting Blood Glucose(mmol/L)	7.50.13±1.30	7.33±1.01	0.041
HbA1c	8.35±1.04	7.60±1.01	<0.001
Cholesterol (mmol/L)	3.40±0.66	3.16±0.80	0.002
HDL-C (mmol/L)	1.45±0.90	1.22±0.37	0.001
LDL-C (mmol/L)	1.97±0.92	1.72±0.69	0.025
Albumin (mmol/L)	40.10±4.41	38.68±4.09	0.001
Bilirubin (mmol/L)	7.10±1.46	6.12±1.64	0.001
Triglyceride (mmol/L)	1.82±0.87	1.72±2.58	0.110
Uric Acid (mmol/L)	275.8±99.8	287.9±89.51	0.272
Systolic Blood Pressure mm Hg	131.7±9.25	127.73±11.98	0.001
Diastolic Blood Pressure mm Hg	82.20±8.60	79.80±8.20	0.004
Microalbuminuria	14.59±2.49	9.07±3.50	<0.001

	**n(**%**)**	**n(**%**)**	

**Vitamin D Level**			
Deficiency 25(OH)D <20 ng/ml	68(54.0)	212(41.4)	
Insufficiency 25(OH)D 20-29 ng/ml	38(30.2)	183(35.7)	0.004
Optimal 25(OH)D 30-80 ng/ml	20(15.8)	117(22.9)	
**Retinopathy**			
Present	24(19.0)	52(10.2)	
Absent	102(81.0)	460(89.8)	0.006
**Visual impairment level**			
None	102(81.0)	460(89.8)	
Mild	9(7.1)	28(5.5)	0.007
Moderate or more	15(11.9)	24(4.7)	

**Table 3:** Mean pure tone thresholds for hearing loss and normal hearing among diabetes subjects (N = 638).

**Variables**		**Retinopathy with ** **Hearing Loss ** **≥26 dB** **n=126** **Mean ± SD**	**Non-Retinopathy ** **Normal Hearing with ** **diabetic <26 dB** **N = 512** **Mean ± SD**	**p-value ** **significance**
**Frequency Side**				
**250 Hz **	Ri	27.03±15.57	22.96±7.22	< 0.001
**250 Hz**	Le	32.54± 16.71	23.20±12.18	< 0.001
**500 Hz**	Ri	33.85±18.32	19.20±14.79	< 0.001
**500 Hz**	Le	29.60±14.54	18.47±12.38	< 0.001
**1,000 Hz**	Ri	31.43±17.58	16.26±9.33	< 0.001
**1,000 Hz**	Le	30.67±17.26	16.31±12.53	< 0.001
**1,500 Hz**	Ri	31.90±18.5	17.21±10.71	< 0.001
**1,500 Hz**	Le	31.34±16.81	16.68±13.24	< 0.001
**2,000 Hz**	Ri	32.38±19.42	18.17±12.10	< 0.001
**2,000 Hz**	Le	32.02±16.36	17.06±13.96	< 0.001
**3,000 Hz**	Ri	33.79±20.62	20.95±12.50	< 0.001
**3,000 Hz**	Le	34.00±17.96	20.38±15.58	< 0.001
**4,000 Hz**	Ri	35.20± 21.82	23.73±12.92	< 0.001
**4,000 Hz**	Le	35.99± 19.56	23.70±17.21	< 0.001
**6,000 Hz**	Ri	38.97±24.83	27.22±17.48	< 0.001
**6,000 Hz**	Le	37.03±20.04	25.35±17.29	< 0.001
**8,000 Hz**	Ri	42.74±27.85	30.71±22.05	< 0.001
**8,000 Hz**	Le	38.08±20.52	27.01±17.37	< 0.001
**PTA**	Ri	33.21±14.55	19.34±10.42	< 0.001
**PTA**	Le	32.07±10.11	19.07±9.89	< 0.001
**SRT**	Ri	35.51±18.44	26.34±12.51	< 0.001
**SRT**	Le	35.87±20.81	27.85±16.34	< 0.001
**SDS (**%**)**	Ri	66.16±14.08	76.00±15.67	< 0.001
**SDS (**%**)**	Le	67.00±10.53	75.17±15.20	< 0.001

**PTA:** pure tone average; **SRT:** speech reception threshold; **SDS:** speech discrimination score.

**Table 4:** Multivariate stepwise logistic regression analysis for predictors of hearing loss and retinopathy among T2DM patients (N=638).

**Variables**	**Adj. OR (95**%**CI)**	**P value**
**Vertigo**	4.09 (1.79-9.66)	<0.001
**Duration of DM**	1.92 (1.70-2.44)	<0.001
**Mobile/I pad Phone use**	4.25 (2.85-9.97)	<0.001
**Vitamin D deficiency**	2.03(1.94-3.06)	0.002
**Sleep disturbance**	2.68 (2.24-3.58)	0.004
**Head ache**	2.39(1.30-4.32)	0.005
**Frequently TV watching**	2.42 (1.26-4.00)	0.006
**Tinnitus**	1.95(1.22-3.13)	0.007
**Cigarette Smoking (yes)**	1.79 (1.26-3.23)	0.029
**Hypertension**	1.71 (1.36-2.82)	0.036

## Data Availability

The data used to support the findings of this study are available from the corresponding author upon request.
